# A pharmacometric multistate model for predicting long-term treatment outcomes of patients with pulmonary TB

**DOI:** 10.1093/jac/dkae256

**Published:** 2024-08-01

**Authors:** Yu-Jou Lin, Yuanxi Zou, Mats O Karlsson, Elin M Svensson

**Affiliations:** Department of Pharmacy, Uppsala University, Uppsala, Sweden; Department of Pharmacy, Uppsala University, Uppsala, Sweden; Department of Pharmacy, Uppsala University, Uppsala, Sweden; Department of Pharmacy, Uppsala University, Uppsala, Sweden; Department of Pharmacy, Radboud University Medical Center, Nijmegen, The Netherlands

## Abstract

**Background:**

Studying long-term treatment outcomes of TB is time-consuming and impractical. Early and reliable biomarkers reflecting treatment response and capable of predicting long-term outcomes are urgently needed.

**Objectives:**

To develop a pharmacometric multistate model to evaluate the link between potential predictors and long-term outcomes.

**Methods:**

Data were obtained from two Phase II clinical trials (TMC207-C208 and TMC207-C209) with bedaquiline on top of a multidrug background regimen. Patients were typically followed throughout a 24 week investigational treatment period plus a 96 week follow-up period. A five-state multistate model (active TB, converted, recurrent TB, dropout, and death) was developed to describe observed transitions. Evaluated predictors included patient characteristics, baseline TB disease severity and on-treatment biomarkers.

**Results:**

A fast bacterial clearance in the first 2 weeks and low TB bacterial burden at baseline increased probability to achieve conversion, whereas patients with XDR-TB were less likely to reach conversion. Higher estimated mycobacterial load at the end of 24 week treatment increased the probability of recurrence. At 120 weeks, the model predicted 55% (95% prediction interval, 50%–60%), 6.5% (4.2%–9.0%) and 7.5% (5.2%–10%) of patients in converted, recurrent TB and death states, respectively. Simulations predicted a substantial increase of recurrence after 24 weeks in patients with slow bacterial clearance regardless of baseline bacterial burden.

**Conclusions:**

The developed multistate model successfully described TB treatment outcomes. The multistate modelling framework enables prediction of several outcomes simultaneously, and allows mechanistically sound investigation of novel promising predictors. This may help support future biomarker evaluation, clinical trial design and analysis.

## Introduction

TB is a considerable public health threat worldwide. In 2022, an estimated 10.6 million people fell ill with TB, and approximately 1.3 million died from the disease, making TB the second deadliest infectious killer after coronavirus disease (COVID-19).^1^ Given that this disease recurs after completion of therapy, studies of long-term treatment outcomes are of crucial importance. In TB studies, microbiological cultures remain the cornerstone for evaluation of treatment efficacy. Several biomarkers [e.g. change in cfu in solid media or time-to-positivity (TTP) in liquid media, sputum culture conversion (SCC)] are well-established outcome measurements in early bactericidal activity studies and clinical trials.^2–5^ To date, however, early culture-based biomarkers have only modest success in predicting long-term outcomes.^6^ In addition, culture positivity is a dichotomous marker and therefore has relatively low statistical power.^7^ Furthermore, the procedures of culture-based metrics are usually time-consuming (up to 6–8 weeks), vulnerable to contamination and labour-intensive.^2^ Long follow-up period, high cost and aforementioned obstacles seriously impede TB drug development. Alternative, reliable, early biomarkers reflecting treatment response and possibly capable of predicting long-term outcomes, are absolutely needed to speed up TB drug development and, further, improve individual patient care.

In TB studies, favourable outcomes refer to treatment success including cured or treatment completion, and unfavourable outcomes refer to treatment failure, death and loss to follow-up.^8^ Classical time-to-event analysis can only focus on one primary outcome while ignoring the influence of intermediate events that happen before an outcome of interest.^9^ A multistate model can describe longitudinal changes during the course of disease and recovery by considering multiple outcomes (i.e. states) and possible transitions between them.^9–11^ Moreover, a multistate model has benefits for evaluation of predictors because the mechanism of the predictor can be better accounted for with a more granular outcome definition.^11,12^ A multistate model in pharmacometric settings is a powerful tool to investigate the potential impact of pharmacokinetic (PK) and pharmacodynamic (PD) model-derived markers on transitions.^13–15^ In this sense, it can help to identify novel biomarkers with the link to individual exposure and response in an early phase to guide treatment strategies. The aim of this study was to develop a multistate model framework for patients with pulmonary TB and to explore the link between long-term outcomes and selected markers of early treatment response.

## Methods

### Clinical study data

Data originated from the TMC207-C208 (NCT00449644) and TMC207-C209 (NCT00910871) Phase II clinical trials sponsored by Janssen Research & Development. The C208 study enrolled patients with MDR-TB receiving either placebo or bedaquiline on top of a predefined anti-TB drug regimen. Patients who had previously received anti-TB treatment were excluded. In the C209 study, patients with either MDR-TB or XDR-TB were enrolled, and all received bedaquiline together with an individualized background regimen. Patients with prior TB treatment before enrollment in the study were eligible. The old definitions of drug resistance TB profiles, i.e. the definition prior to 2021, from WHO were used.

In both studies, bedaquiline dosage was 400 mg once daily for the first 2 weeks, followed by 200 mg three times a week for a further 22 weeks (or 6 weeks for C208 stage 1 study). All patients were followed until 120 weeks (104 weeks for the C208 stage 1 study). Sputum samples in triplicates were collected in liquid medium using Mycobacteria Growth Indicator Tubes at each scheduled visit. TTP was recorded for 42 days, and cultures without signals beyond 42 days were classified as negative. For the C208 study, sputum cultures were sampled every week until Week 8, and then every other week until Week 24. Additional sputum cultures were collected at Weeks 28, 32, 36, 48, 60, 72, 84, 96, 108 and 120 throughout the 96 week follow-up period (sampled at Week 104 instead of Week 108 and Week 120 for the C208 stage 1 study). For the C209 study, sputum samples were taken at each scheduled visit at Weeks 2, 4, 8, 12, 16, 20 and 24, and then every 12 weeks until 120 weeks. Both studies were conducted according to Good Clinical Practice. Ethical approval for the two trials was obtained from independent local ethics committee and regulatory boards. Detailed study designs are described in the Supplementary material (available as Supplementary data at *JAC* Online) and original publications.^16,17^

### Pharmacometric multistate modelling

The developed multistate model framework for pulmonary TB was composed of five states:

S1—Active TB (active tuberculosis after initial infection);
S2—Converted (the day of the first of two negative sputum cultures collected at least 25 days apart, not intervened by positive cultures, i.e. no detectable active infection);
S3—Recurrent TB (the day of the first of two consecutive positive sputum cultures or a single positive result before the patient completed or discontinued from the trial, after conversion);
S4—Dropout (discontinued study visits for any reason except death before the last scheduled visit);
S5—Death.

The definitions of S2 and S3 were based on sputum culture status throughout the study period. Therefore, patients who had bacteriological reversion (i.e. cultures returned to positive after conversion) whether on treatment or after treatment completion were both defined as in S3 in the developed multistate model.

In our analysis, all patients were assigned to the active TB state (S1) at treatment initiation. During the study, patients could either stay in the current state or transit to different states. Patients with active TB could have sputum conversion (i.e. move to S2) and further develop recurrence (i.e. move to S3). Those in S3 who reached sputum conversion again moved back to S2. Patients could drop out (S4) from the study or die (S5) from any state. Transition rates λ*_ij_* were used to describe the hazard from state *i* to state *j* and estimated based on the observed data. λ_12_ and λ_23_ represent conversion and recurrence, respectively. Parametric hazard functions including constant and Weibull distributions were tested for each transition rate. A symmetric surge function was tested for transition λ_12_ based on the previous findings.^18,19^

Potential predictors explored on each transition rate included baseline patient characteristics and on-treatment biomarkers. Investigated baseline covariates included demographics (age, sex, weight, investigator-reported race, country), drug resistance profile of TB, concomitant HIV infection [Yes/No (Y/N)], the presence of cavitation (Y/N), baseline TTP (mean TTP value of triplicates), bedaquiline treatment (Y/N), previous anti-TB treatment before being enrolled in the study (Y/N), and study (C208/C209). Post-baseline time-varying predictors were derived from the previous developed PKPD models,^20–22^ including albumin levels, body weight change, model-derived mycobacterial load (MMBL) and half-life of bacterial clearance (HL). MMBL and HL are model-derived on-treatment biomarkers integrating information of baseline bacterial load, prior anti-TB treatment, drug resistance profile, and individual drug exposure over time. Additionally, biomarkers of interest such as time to SCC, culture status at Month 2, and MMBL at the end of 24 week treatment (MMBL_end_) were evaluated on transitions from converted or recurrent TB states. For handling missing covariate values, the median was used for continuous variables and mode was used for categorical variables.

The model development and covariate selection were based upon likelihood-ratio testing through a stepwise procedure at significance levels of 0.05 and 0.01 for forward inclusion and backward elimination, respectively, parameter precision determined by the variance-covariance matrix and summarized as relative standard error, and visual predictive checks. To evaluate model predictive performance on an individual level, the Brier score was used.^13,23,24^ A lower Brier score implies better model performance in forecasting an event of interest. The Brier skill score, which is based on a comparison of Brier scores, can quantify the degree of improvement in probabilistic predictions from a reference model; therefore, it enables inter-model comparison and evaluation of the impact of predictors. A positive Brier skill score indicates better predictive performance of a new model compared with a reference model. Details on model equations, predictors investigation and all software used are provided in the Supplementary material.

### Simulation scenarios

To investigate the influence of identified predictors on possible outcomes, scenarios with various TB disease severity (e.g. baseline TTP, type of resistance profile) and on-treatment biomarkers (e.g. HL, MMBL) based on relevant covariates identified in the multistate model were selected. The observed 5th, 50th and 95th percentiles of the continuous covariates and presented categories of the categorical covariates of the interest were used. Other covariates not investigated were set to be the same value as a reference individual. For each scenario, simulations were performed for a set of 10 000 virtual patients and repeated 1000 times with sampled parameter vectors from the variance-covariance matrix to account for parameter uncertainty. The proportions of patients in each state at 8, 24, 48, 72 and 120 weeks were summarized.

## Results

### Patient population and characteristics

Of the 439 patients from the C208 and C209 studies, 35 participants having negative cultures at screening and baseline and 2 participants with only observations before treatment initiation were excluded. Of the total 402 patients included in the analysis, 19 patients had missing information of sputum cultures at baseline and were assumed to have active TB disease in the beginning. Baseline patient characteristics are summarized in Table 1.

**Table 1.** Patient characteristics in the dataset (*n* = 402) used for multistate model development

Characteristic	Median (range) or *n* (%)
Age,^a^ y	33 (18–68)
Weight at baseline,^a^ kg	55 (30–113)
Sex^a^	
Male	262 (65)
Female	139 (35)
Type of drug-resistant TB^b^	
Drug-sensitive	11 (3)
MDR	185 (54)
Pre-XDR	95 (28)
XDR	48 (14)
Study	
C208	195 (49)
C209	207 (51)
Participants receiving bedaquiline treatment	303 (75)
Previous anti-TB treatment	186 (46)
Participants living with HIV^c^	36 (9)
Presence of lung cavitation^a^	385 (96)
From a high TB burden country^d^	300 (75)
Mean TTP at baseline,^e^ d	9.1 (2.3–42)

TTP, time-to-positivity.

^a^One missing value.

^b^Sixty-three missing values. The old definition (prior to 2021) of MDR-TB, pre-XDR-TB and XDR-TB was used in the analysis.

^c^Eight missing values.

^d^The definition of a high TB burden country is according to WHO’s Global Tuberculosis Report.

^e^Nineteen missing values. Mean TTP represents the mean value of triplicate sputum cultures.

### Multistate model

**Figure 1.**
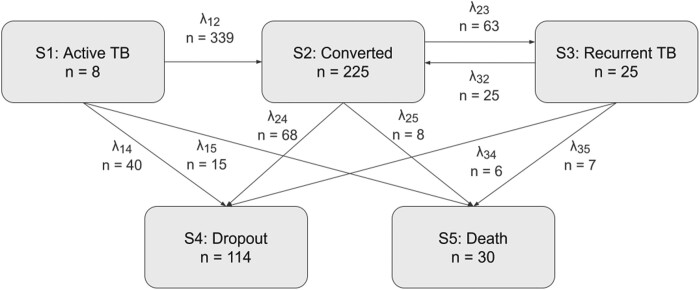
A schematic diagram of the multistate model in patients with pulmonary tuberculosis. The number in each state indicates the number of patients staying in the state at the end of study. The number on each transition rate indicates the total of transition events observed throughout the 120 week study (a 24 week investigational treatment period followed by a 96 week follow-up period). λ*_ij_* describes the transition rate from state *i* to state *j*. Note that λ_12_ represents conversion, and λ_23_ represents recurrence.

Figure 1 shows a schematic diagram of the multistate model framework for pulmonary TB and the number of observed transitions between states. Of 6984 observations from 402 patients, there were 364 conversion events (25 had sputum conversion again after experiencing recurrence) and 63 recurrence events throughout the analysis. Thirty patients died, and a total of 114 patients had dropped out by the end of study. The transition rate λ_12_ (conversion) followed a surge function described by the surge amplitude (SA), peak time (PT) and surge width (SW) parameters. λ_12_ was estimated to reach the highest peak at 11.4 weeks (95% CI, 10.0–12.8 weeks). The transition rates λ_23_ (recurrence) and λ_32_ (from recurrent TB state to converted state) were constant over time. The transitions of dropout from any state [active TB (λ_14_), converted (λ_24_) and recurrent TB (λ_34_)] were all constant. λ_24_ and λ_34_ were set to the same value as no significant difference was found when they were estimated separately. Similarly, the transition hazards from active TB and recurrent TB to death (λ_15_, λ_35_) were set to the same. Both λ_15_ and λ_35_ followed a Weibull distribution with increasing hazard over time, whereas the transition hazard from converted to death (λ_25_) was constant.

### Predictors of state transitions

**Figure 2.**
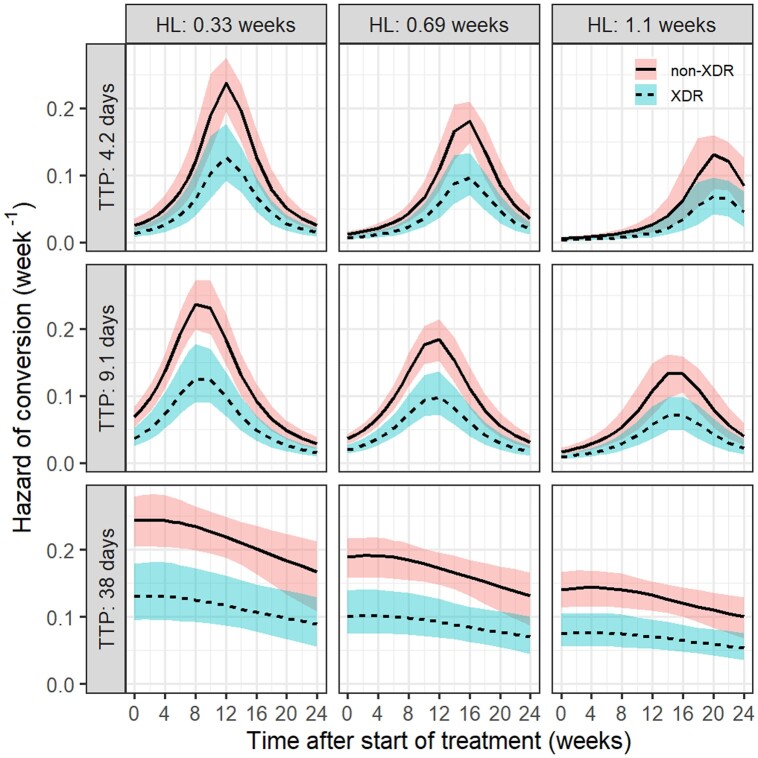
The surge hazard function of conversion over time until 24 weeks stratified by HL at Week 2 (5th, 50th and 95th percentiles), baseline TTP (mean TTP value of triplicates, 5th, 50th and 95th percentiles) and type of drug resistance profile (non-XDR in solid lines and XDR in dashed lines). Lines depict the median values of the hazard with the combination of predictors, shaded areas represent the 90% CI considering uncertainties of the estimated parameters. HL, half-life of bacterial clearance; TTP, time-to-positivity. This figure appears in colour in the online version of *JAC* and in black and white in the print version of *JAC*.

Model-derived half-life of bacterial clearance up to Week 2 (HL_2_), mean TTP at baseline, and type of drug-resistant TB were jointly significant predictors of conversion (λ_12_). The impact of the predictors on λ_12_ is illustrated in Figure 2. Patients with a shorter HL_2_ (i.e. faster bacterial clearance) and longer baseline TTP (i.e. lower bacterial burden) were more likely to achieve conversion. For patients with XDR-TB, λ_12_ decreased by 46% (95% CI, 24%–62%), indicating that the likelihood of achieving conversion for these patients was lower compared with those with non-XDR-TB. Details about predictors on the surge function parameters of λ_12_ are available in Supplementary material.

**Figure 3.**
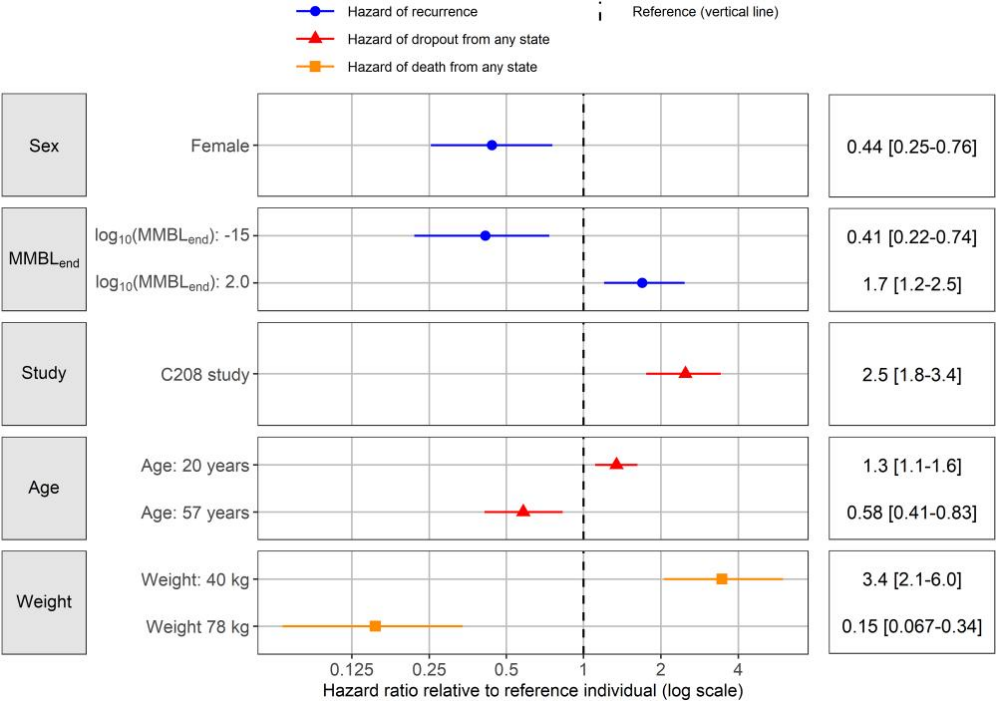
Predictor impacts on the hazards of recurrence, dropout from any state and death from any state. The median HRs of recurrence (shown in circles), dropout from any state (shown in triangles), and death from any state (shown in squares) relative to the reference individual with 90% CI (shown in horizontal bars) are represented. The dotted vertical line indicates the equal hazard compared with reference individual (HR = 1). The 5th and 95th percentiles of values for log_10_(MMBL_end_), age and weight were used to assess the effect of predictors on hazards. The reference individual is a 33-year-old male weighing 55 kg, infected with MDR tuberculosis, enrolled in the C209 study, 9.1 days of baseline time-to-positivity, 0.69 weeks of half-life of bacterial clearance at Week 2, and −4.3 log_10_(MMBL_end_). MMBL_end_, model-derived mycobacterial load at the end of 24 week treatment. This figure appears in colour in the online version of *JAC* and in black and white in the print version of *JAC*.

Being male was significantly correlated with higher risk of recurrence (λ_23_), and having higher MMBL at the end of 24 week treatment (MMBL_end_) increased λ_23_ after 24 weeks. Participants enrolled in the C208 study and of younger age had a higher risk of dropping out from any state. Patients with lower baseline weight were found to have a higher risk of dying. The impact of predictors on each transition hazard except λ_12_ is displayed in Figure 3. The presence of cavitation and HIV status were not identified as predictors of any transition hazard between each state. Likewise, no correlations between short-term treatment responses such as time to SCC and Month 2 culture status and hazards to recurrent TB, dropout and death were found. Statistical significance for each identified predictor is presented in Table S1.

### Model evaluation

**Figure 4.**
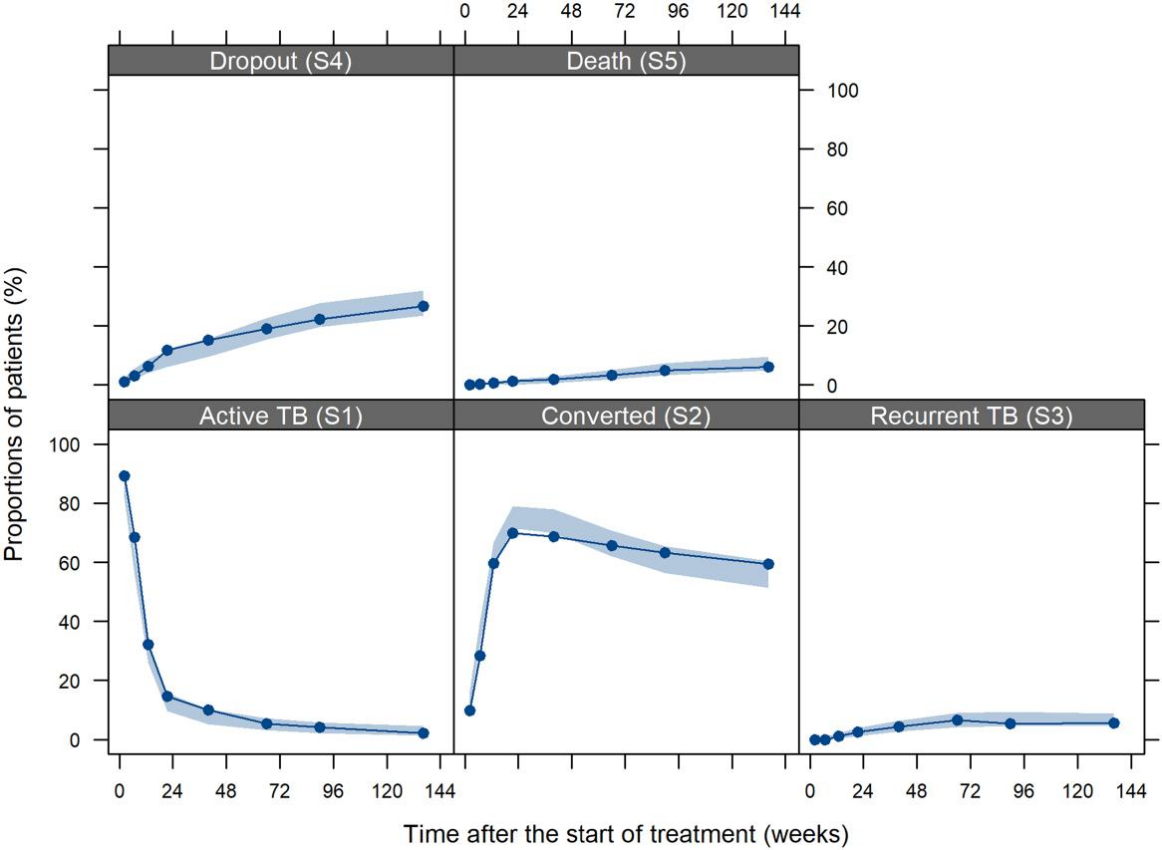
Visual predictive checks of proportions of patients in each specified state over time. Solid lines represent the observed proportions of patients in different states, and shaded areas represent the 95% prediction intervals from 1000 simulated replicates. This figure appears in colour in the online version of *JAC* and in black and white in the print version of *JAC*.

The final parameter estimates are available in Table S2. The final multistate model described the overall observed transitions well (Figure 4 and Figure S1). The predicted proportions of patients at 120 weeks in converted, recurrent TB and death states were 55% [95% prediction interval (PI), 50%–60%], 6.5% (95% PI, 4.2%–9.0%) and 7.5% (95% PI, 5.2%–10%), respectively.

**Figure 5.**
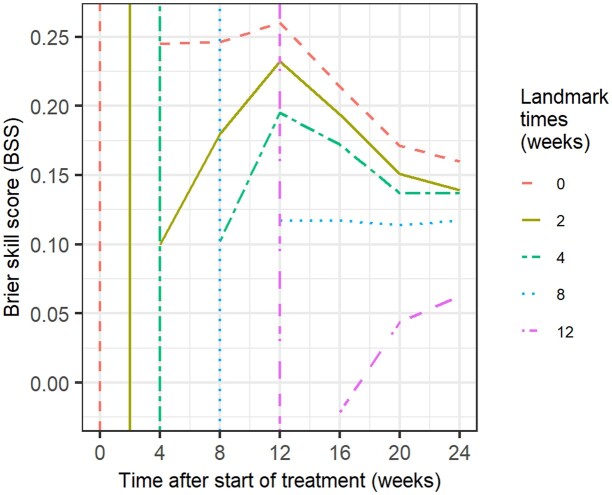
The Brier skill scores (BSS) for assessing the predictive performance of the final multistate model with various landmark times in forecasting the individual probability of conversion. A positive value of Brier skill score indicates the degree of relative improvement of final multistate model in predictive accuracy compared with the base model (with no covariates). This figure appears in colour in the online version of *JAC* and in black and white in the print version of *JAC*.

The predictive performance of the final multistate model based on varying durations of data collection (i.e. varying landmark time) was evaluated using the Brier score. Overall, Brier scores in the final multistate model were lower (i.e. better) compared with the base model, in which no predictors were included (Tables S3 and S4). During the 24 week treatment period, Brier skill scores in forecasting conversion were positive all the time and reached a peak at 12 weeks (Figure 5), indicating that the final model performed better (up to 26% improvement) compared with the base model. Of note, the degree of improvement was decreasing when landmark time was longer. In terms of recurrence, Table S4 demonstrates the Brier score was generally low and Brier skill scores were near 0 across different landmark times.

In order to investigate the influence of the time-varying bacterial clearance HL_2_, Brier score at landmark time = 2 weeks of the model without this predictor was calculated while keeping other predictors the same as the final model. A 5%–10% improvement was observed during the first 24 weeks when HL_2_ was included (Table S3).

### Simulation scenarios

**Figure 6.**
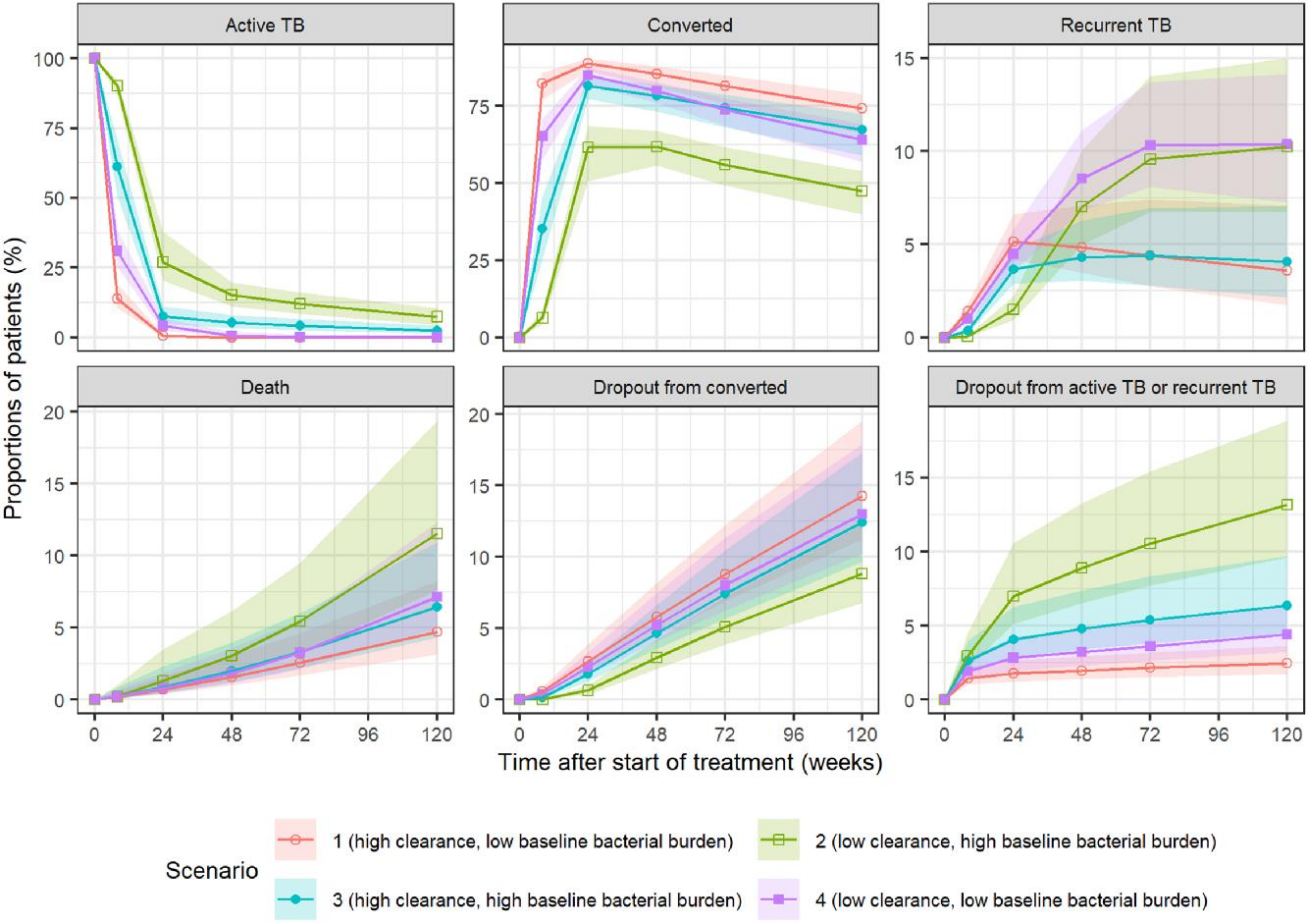
The proportions of patients in selected scenarios with possible outcomes throughout the study. Lines with different symbols indicate the median of predicted proportions of patients in each state under selected scenarios. Shaded areas indicate the 90% prediction intervals representing parameter estimate uncertainty. Bacterial clearance was represented by half-life at Week 2, and bacterial burden was represented by time-to-positivity at baseline. High and low values represent the 5th and 95th percentiles of the corresponding parameters (high clearance: 0.33 weeks of half-life at Week 2; low clearance: 1.1 weeks of half-life at Week 2; high baseline bacterial burden: 4.2 days of baseline time-to-positivity; low baseline bacterial burden: 38 days of baseline time-to-positivity), respectively. Except for half-life and time-to-positivity, patients in every scenario were assumed to have the same values of other predictors as a reference individual. This figure appears in colour in the online version of *JAC* and in black and white in the print version of *JAC*.

Scenarios for patients with varying extent of TB disease severity and early treatment response were selected [jointly represented by HL (i.e. bacterial clearance), TTP at baseline (i.e. bacterial burden), presence of XDR-TB resistance). Figure 6 shows the proportions of patients with different treatment outcomes over 120 weeks. At 24 weeks, the proportions of patients who reached SCC ranged from 62% (90% PI, 51%–69%) (the most severe scenario) to 89% (90% PI, 86%–90%) (the least severe scenario). A substantial increase of recurrence in low bacterial clearance groups was observed after 24 weeks, irrespective of baseline bacterial burden. Simulations comparing participants with or without XDR-TB are shown in Figure S2.

## Discussion

In this study, we developed a multistate model for TB treatment outcomes and explored potential predictors describing the transitions between states during treatment and follow-up periods. Conversion (λ_12_) was best described by a surge function; this aligns with prior research in modelling time to SCC.^18,19^ Baseline TTP is recognized as an influential predictor of culture conversion,^3,21,25^ and our analysis showed a similar correlation between a longer baseline TTP (i.e. lower bacterial burden) and faster conversion. Low model-derived half-life of bacterial clearance in the first 2 weeks (HL_2_) was found to increase the amplitude of surge peak and move the peak time forward for λ_12_, suggesting that bactericidal activity in the early phase is important to achieve SCC. Such an indicator representative of the bacterial kill rate has been used in another study,^26^ where a slope suggested to represent kill of non-replicating *Mycobacterium tuberculosis* derived from 8 week TTP data was found capable of predicting long-term clinical endpoints. Our study also demonstrated that patients with XDR-TB were less likely to achieve SCC, which is in line with previous studies.^22,27^

We found that the risk of recurrence was higher for men, and this finding has been reported earlier in several studies.^28,29^ One possible explanation is that male patients were perceived as being more non-adherent to anti-TB drugs, and this might lead to a poor treatment outcome.^30,31^ Notably, model-derived mycobacterial load at the end of treatment (MMBL_end_) was identified as a significant predictor of recurrence after the completion of 24 week treatment. We hypothesize that MMBL_end_ represents a composite of disease severity at baseline and on-treatment response throughout the whole treatment period. Nevertheless, the results of Brier skill scores show little improvement in forecasting recurrence when MMBL_end_ was included. Additional studies are needed to evaluate the validity of using MMBL_end_ for predicting recurrence.

The dropout rate in patients from the C208 study was higher than in C209, and this might be explained by different trial designs and time effect. Unlike the open-label single-arm C209 trial, patients in a double-blind, placebo-controlled trial like C208 might be less willing to return for scheduled follow-ups with few signs of improvement.^32^ Additionally, C209 was conducted after positive results for bedaquiline had been reported for C208. Patients in C209 might therefore have higher confidence in drug efficacy, leading to lower dropout rate. The correlation between younger age and increasing risk of dropout might be related to job demands for workers and less attendance for the scheduled visit.^33^ A common symptom of TB is loss of appetite and unintentional weight loss. In our study, lower weight at baseline was correlated with a higher risk of death, implying the pretreatment TB disease severity was a determinant of an unfavourable TB outcome. Nevertheless, this finding should be interpreted with caution outside the investigated weight range.

Time to SCC in previously treated patients has been shown to be longer than in those who were treatment-naive.^3,22^ Although receiving anti-TB therapy or not prior to the study was not found to be a predictor of any transitions in this analysis, the information was indirectly included through the bacterial clearance and MMBL metrics.^22^ The impacts of cavitary disease and concomitant HIV infection on treatment outcomes of TB have been established in some studies^3,25,34^ but not in our current analysis. A possible explanation might be related to the fact that the majority of patients in this analysis had cavitary disease (96%) and were not living with HIV (91%).

The Brier skill scores showed that predictive ability of the final multistate model improved the most at start of treatment (landmark time = 0). As data were collected longer (i.e. longer landmark time), baseline patient characteristics only had moderate effects on improving the forecast for conversion. However, in forecasting recurrence Brier skill scores did not show the same trend; they remained nearly 0 in the whole study period. One possible explanation is that this performance metric cannot sufficiently discriminate models while forecasting a rare event,^35^ given that the recurrence rate in our analysis was only around 6% at 2 years. Secondly, the predictor MMBL_end_ was rather weak (*P* value = 0.0061, see Table S1), which might lead to little improvement on predicting recurrence events.

The simulations showed that patients with low baseline bacterial burden tended to achieve SCC earlier regardless of the rate of clearance; this agrees with previous findings.^36^ Notably, high baseline bacterial load is not a decisive factor of SCC by the end of treatment as long as the ability to clear mycobacteria is high. In contrast to conversion, low MMBL_end_ was found mainly to be driven by the rate of bacterial clearance, indicating bacterial clearance is a crucial factor of recurrence other than pretreatment bacterial burden. This is physiologically plausible since relapse is found associated with sterilizing activity, which represents the terminal bacterial kill rate.^26,37^

There are several limitations in our model. First, relapse caused by the original TB strain and exogenous reinfection could not be distinguished due to the lack of genotyping results. In this sense the model cannot differentiate the predictor effects on relapse and reinfection. Nevertheless, it has been reported that relapses predominantly occur within 1 year after treatment completion.^38^ In addition, more reinfection cases are identified in countries with high TB disease burden,^39^ whereas country was not identified as a predictor in our study. Hence, it is reasonable to assume that the recurrence cases in our work are mainly relapses. Second, the metric HL_2_ was used to explain the transition hazard λ_12_, implying that individual drug exposures after Week 2 did not affect the probability of SCC. Nonetheless, the longitudinal individual drug exposure over the 24 week treatment period was taken into account while computing MMBL_end_, indicating its impact on predicting recurrence events. Third, approximately 16% of patients had missing information on drug resistance TB profiles. As no significant difference was identified while separating these patients from the MDR-TB category, those with missing information were assigned to the MDR-TB group. Lastly, there are several potential predictors not explored in our study due to the limited data. For example, host immunological biomarkers have been found to be capable of distinguishing treatment outcomes.^40,41^ Some studies have postulated that cell wall lipid composition in *M. tuberculosis* subpopulations is relevant to drug resistance profiles.^42,43^ A novel molecular marker, ribosomal RNA synthesis (RS) ratio, can be used to evaluate drug sterilizing activity.^44^

The current existing prediction models were mostly developed for a single or composite TB outcome.^45,46^ Our multistate model enables simultaneous evaluation of several TB treatment outcomes and mechanistically sound investigation of novel promising predictors. The model can also be used for simulating outcomes in different scenarios, allowing development of stratified medicine strategies.^47^ A similar model structure is expected to be applicable to other TB treatment regimens by re-estimating actual hazards and re-examining identified covariates. However, further validation on independent datasets to demonstrate the generalizability of the structure and hazard shapes would be beneficial.^45^

To the best of our knowledge, this is the first multistate model framework estimating treatment effects in the TB disease and recovery process. The developed multistate model successfully described treatment outcomes over more than 2 years. This approach may help support future biomarker evaluation, trial design and analysis with the major objective of facilitating TB drug development and individual care.

## Supplementary Material

dkae256_Supplementary_Data
