# Supplementary material for: A pharmacometric multistate model for predicting long-term treatment outcomes of patients with pulmonary TB
Source: J Antimicrob Chemother. 2024 Aug 1;79(10):2561–9. doi: 10.1093/jac/dkae256 (PMC11441995; doi:10.1093/jac/dkae256)
Supplement: dkae256_Supplementary_Data [file dkae256_supplementary_data.pdf]

Supplementary Data

A pharmacometric multistate model for predicting long-term treatment outcomes of patients with pulmonary tuberculosis

Yu-Jou Lin[1], Yuanxi Zou[1], Mats O. Karlsson[1], Elin M. Svensson[1,2]

[1]Department of Pharmacy, Uppsala University, Uppsala, Sweden

[2]Department of Pharmacy, Radboud University Medical Center, Nijmegen, The Netherlands

Correspondence:

Elin M. Svensson

Phone: +46 18-471 4105

Address: Department of Pharmacy, Uppsala University, P.O. Box 580, 751 23 Uppsala, Sweden

Email: elin.svensson@farmaci.uu.se

Table of Contents

Study design 3

Multistate model 4

Model equations 4

Predictors investigation 5

Software 6

Results of multistate modeling 7

Impact of predictors 7

Final parameter estimates 8

Model evaluation 9

Simulations in the population with XDR-TB infection 11

NONMEM code 12

References 17

Study design

The TMC207-C208 study (ClinicalTrials.gov number NCT00449644) was a randomized, double-blind, placebo-controlled, Phase IIb trial.[1] Patients from Brazil, India, Latvia, Peru, Philippines, Russia, South Africa, and Thailand were recruited. The C208 study enrolled patients with newly-diagnosed MDR-TB to evaluate the efficacy, safety, and tolerability of bedaquiline. Participants were receiving either bedaquiline or placebo on top of a five-drug background therapy (pyrazinamide, ofloxacin, kanamycin, ethionamide, and cycloserine, changes allowed based on adverse events or drug susceptibility testing if necessary). The C208 study consisted of 2 stages: in the stage 1, the placebo/bedaquiline treatment duration was 8 weeks, while in the stage 2 the duration was 24 weeks. Patients receiving previous anti-TB treatment before enrolled in the study or infected by HIV with a CD4+ count less than 300 cells/mm^3 were excluded in this study.

The TMC207-C209 study (ClinicalTrials.gov number NCT00910871) was an open-label, single-arm Phase IIb trial, and the trial sites were in 11 countries including China, Estonia, Latvia, Peru, Philippines, Russia, Turkey, Ukraine, South Africa, South Korea, and Thailand.[2] The C209 study enrolled patients with both newly-diagnosed and treatment-experienced MDR-TB under treatment of bedaquiline for 24 weeks in combination with an individualized background therapy selected by investigator according to guidelines for National Tuberculosis Program. Patients with XDR-TB were eligible if they did not develop resistance to at least 3 medications in their background therapy. Patients could be treated with anti-TB drugs before the start of bedaquiline treatment, and patients having a positive result of HIV test with a CD4+ count above than 250 cells/mm^3 were eligible.

In both trials, the background MDR-TB treatment regimen continued for the following 12-18 months (48-72 weeks) according to the national TB treatment guidelines after the completion of bedaquiline treatment. Modifications of the background regimen were allowed according to the current standard clinical practice, susceptibility test results as well as discussions with medical leaders.

Multistate model

Model equations

The probability that a transition occurs over time can be described by the differential equations shown below (Equations 1-5):

$$S1_0 = 1$$

$$\frac{dS_1}{dt} = -S1 * (\lambda_{12} + \lambda_{14} + \lambda_{15}) \quad (1)$$

$$S2_0 = 0$$

$$\frac{dS_2}{dt} = S1 * \lambda_{12} + S3 * \lambda_{32} - S2 * \lambda_{23} - S2 * \lambda_{24} - S2 * \lambda_{25} \quad (2)$$

$$S3_0 = 0$$

$$\frac{dS_3}{dt} = S2 * \lambda_{23} - S3 * \lambda_{32} - S3 * \lambda_{34} - S3 * \lambda_{35} \quad (3)$$

$$S4_0 = 0$$

$$\frac{dS_4}{dt} = S1 * \lambda_{14} + S2 * \lambda_{24} + S3 * \lambda_{34} \quad (4)$$

$$S5_0 = 0$$

$$\frac{dS_5}{dt} = S1 * \lambda_{15} + S2 * \lambda_{25} + S3 * \lambda_{35} \quad (5)$$

Where Si_0 is the initial probability at time 0 in the state *i*, λ_{ij} the hazard from state *i* to state *j* over time.

Parametric hazard functions including constant and Weibull distributions (Equations 6 and 7) were tested for each transition rate. A symmetric surge function (Equation 8) was tested for transition rate λ_{12} (conversion) based on previous findings.[3,4]

$$\lambda_{ij}(t) = \lambda \quad (6)$$

$$\lambda_{ij}(t) = \lambda\alpha(\lambda t)^{\alpha-1} \quad (7)$$

$$\lambda_{ij}(t) = \frac{SA}{(\frac{t-PT}{SW})^2 + 1} \quad (8)$$

Where $\lambda_{ij}(t)$ is the hazard from state *i* to state *j* over time *t*, λ the scale parameter, α the shape parameter, SA the surge amplitude, PT the peak time, SW the surge width.

Predictors investigation

Post-baseline time-varying predictors (albumin levels, body weight change, half-life of bacterial clearance [HL_t] and model-derived mycobacterial load [$MMBL_t$]) were derived from the previous developed pharmacokinetic (PK) and pharmacodynamic (PD) models.[5–7] The subscript t denotes up to which timepoint the data were used. The metrics explored on each transition were derived based on the observations up to week 2, 4, 8, 24, and investigated prospectively in the multistate model to avoid immortal time bias. That is, only data until time t were used to compute model-derived predictors at time t, and they could only be used to predict outcomes after time t. Therefore, the metrics of an individual were changing over time until time t, and the last observation at time t was carried forward after time t.

In the PKPD model used to derive post-baseline predictors, patients with pre-XDR or XDR-TB infection, less bedaquiline exposure quantified by weekly average concentration, and received anti-TB treatment before start of bedaquiline therapy (for patients from the C209 study) were found to have longer HL of bacterial clearance, indicating their low ability of clearing bacteria. MMBL at treatment initiation was informed by baseline TTP, and the decline of MMBL were determined by HL.[6,7] Weight and albumin levels over time were derived from a previous established bedaquiline PK model.[5] With the information of the baseline body weight and albumin level, a linear model was used to compute individual body weight changing over time, and changes in albumin levels were computed from a self-limiting logistic model. Detailed model structure and development can be found in original publications.

Predictors were generally tested assuming proportional hazards with a baseline transition rate (Equation 9).

$$\lambda_{ij,p} = \lambda_{ij} * e^{\left(\beta * (X_p - X_{median})\right)} \qquad (9)$$

Where $\lambda_{ij,p}$ describes the transition rate of an individual p, λ_{ij} the baseline transition rate, β the coefficient of a predictor, X_p the value of a predictor for individual p. The median value of a predictor X_{median} is introduced only if the predictor is a continuous variable.

By using proportional hazards for investigating predictors, the hazard ratio of a predictor relative to its reference value can be expressed by e^{β} with a unit of increase for a continuous variable or a category of a given categorical variable. When a hazard is described by a surge function, predictors are investigated on each surge parameter (surge amplitude [SA], peak time [PT], and surge width [SW]) separately, and it is worth noting that the impact of a predictor can be evaluated via hazard ratio only when a predictor is introduced on SA parameter.

Each predictor was tested on each transition rate or each surge parameter if a transition was found best described by a surge function. If the same predictor on different transition rates or surge parameters while being estimated separately did not have significant difference, they would collapse into a single estimate.

Software

The modeling was conducted in the NONMEM nonlinear mixed effects modeling software (version 7.5.1).[8] The exact likelihood method was used in model development. Perl-speaks-NONMEM (PsN)[9] was used for executing and handling NONMEM runs. The computations were enabled by resources in project SNIC 2022/22-292 and NAISS 2023/22-1058, provided by the National Academic Infrastructure for Supercomputing in Sweden (NAISS) and the Swedish National Infrastructure for Computing (SNIC) at Uppsala Multidisciplinary Center for Advanced Computational Science (UPPMAX). Data management, graphical analysis and diagnostic plots were performed in R (version 4.2.1)[10] and partially with *Xpose* package (version 4.7.2)[11] and *coveffectsplot* package.[12,13] Simulations with different scenarios were carried out in the R *mrgsolve* package.[14]

Results of multistate modeling

Impact of predictors

Table S1. Univariate deletion from the final multistate model.

Deleted parameter	ΔOFV	Degree of freedom	*p*-value
Mean TTP at baseline on PT_{12}, SW_{12}	122	1	< 0.001
HL_2 on SA_{12}, PT_{12}	44.7	1	< 0.001
XDR-TB on SA_{12}	12.7	1	< 0.001
Sex on λ_{23}	22.5	1	< 0.001
$MMBL_{end}$ on λ_{23}	7.51	1	0.0061
Study (C208/C209) on $\lambda_{14/24/34}$	19.7	1	< 0.001
Age on $\lambda_{14/24/34}$	7.67	1	0.0056
Baseline weight on $\lambda_{15/25/35}$	7.11	1	0.0077

λ_{ij}, the transition rate from state *i* to state *j*. Abbreviations: HL_2, model-derived half-life of bacterial clearance with a dynamic change up to week 2; $MMBL_{end}$, model-derived mycobacterial load at the end of 24-week treatment; OFV: objective function value; PT: peak time; SA: surge amplitude; SW: surge width. TTP, time-to-positivity.

Final parameter estimates

Table S2. The final parameter estimates of the multistate model.

Parameter	Estimates (RSE, %)
SA_{12}, week^{-1}	0.190 (9.5)
PT_{12}, week	11.4 (6.4)
SW_{12}, week	5.59 (12)
λ_{23}[a], week^{-1}	0.00339 (14)
λ_{32}, week^{-1}	0.0162 (21)
λ_{14}, week^{-1}	0.00384 (21)
$\lambda_{24/34}$, week^{-1}	0.00150 (18)
λ_{25}, week^{-1}	0.000263 (38)
$Scale_{15/35}$, week^{-1}	0.00520 (18)
$Shape_{15/35}$	1.96 (19)
$\beta_{HL2\ on\ SA12,\ PT12}$[b]	-0.686 (16)
$\beta_{basTTP\ on\ PT12,\ SW12}$[b]	0.443 (20)
$\beta_{XDR\text{-}TB\ on\ SA12}$[b]	-0.623 (29)
$\beta_{sex\ on\ \lambda23}$[b]	-0.813 (41)
$\beta_{MMBLend\ on\ \lambda23}$[b]	0.0371 (41)
$\beta_{study\ on\ \lambda14/24/34}$[b]	0.910 (22)
$\beta_{age\ on\ \lambda14/24/34}$[b]	-0.0230 (38)
$\beta_{basWT\ on\ \lambda15/25/35}$[b]	-0.0838 (26)

a: λ_{ij}, the transition rate from state *i* to state *j*. b: the coefficient of a predictor. Abbreviations: basWT: body weight at baseline; HL2, model-derived half-life of bacterial clearance with a dynamic change up to week 2; MMBLend, model-derived mycobacterial load at the end of 24-week treatment; PT: peak time; SA: surge amplitude; SW: surge width. basTTP, time-to-positivity at baseline.

Model evaluation

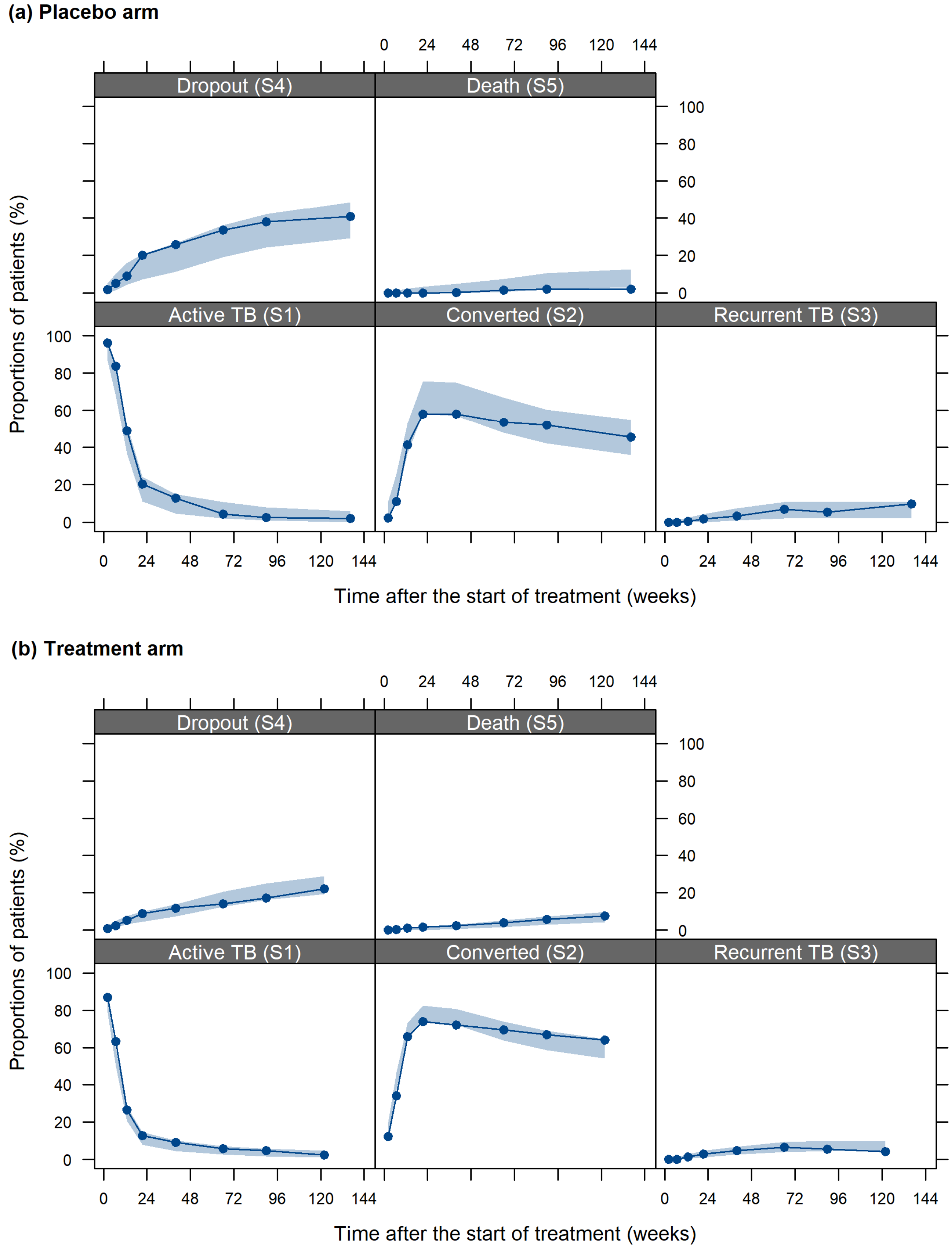

Figure S1. Visual predictive checks of proportions of patients in each specified state over time, stratified by (a) placebo arm and (b) treatment arm. Solid lines represent the observed proportions of patients in different states, and shaded areas represent the 95% prediction intervals from 1,000 simulated replicates.

Table S3. Brier score (BS) and Brier skill score (BSS) for different landmark time in weeks (s) and predicted time points of interest in weeks (t) of base model (no predictors), final model (all predictors), and final model without dynamic model-derived half-life of bacterial clearance up to 2 weeks (HL_2) included (marked as No HL_2 in the table) in forecasting the risk of conversion.

Landmark time (weeks)		s = 0		s = 2		s = 4		s = 8		s = 12		s = 2	
Predicted time (weeks)		Base	Final	Base	Final	Base	Final	Base	Final	Base	Final	No HL_2	Final
t = 4	BS	0.139	0.105	0.070	0.063							0.066	0.063
	BSS	0.245		0.100								0.045	
t = 8	BS	0.244	0.184	0.228	0.187	0.205	0.184					0.201	0.187
	BSS	0.246		0.180		0.102						0.070	
t = 12	BS	0.242	0.179	0.246	0.189	0.246	0.198	0.196	0.173			0.210	0.189
	BSS	0.260		0.232		0.195		0.117				0.100	
t = 16	BS	0.196	0.154	0.206	0.166	0.215	0.178	0.240	0.212	0.189	0.193	0.184	0.166
	BSS	0.214		0.194		0.172		0.117		-0.021		0.098	
t = 20	BS	0.164	0.136	0.172	0.146	0.182	0.157	0.219	0.194	0.227	0.217	0.161	0.146
	BSS	0.171		0.151		0.137		0.114		0.044		0.093	
t = 24	BS	0.144	0.121	0.151	0.130	0.161	0.139	0.197	0.174	0.221	0.207	0.140	0.130
	BSS	0.160		0.139		0.137		0.117		0.063		0.071	

Abbreviations: BS: Brier score; BSS: Brier skill score; HL_2, model-derived half-life of bacterial clearance with a dynamic change up to week 2.

Table S4. Brier score (BS) and Brier skill score (BSS) for different landmark time in weeks (s) and predicted time points of interest in weeks (t) of base model (no predictors) and final model (all predictors) in forecasting the risk of recurrence.

Landmark time (weeks)		s = 0		s = 2		s = 4		s = 8		s = 12		s = 16		s = 24	
Predicted time (weeks)		Base	Final	Base	Final	Base	Final	Base	Final	Base	Final	Base	Final	Base	Final
t = 48	BS	0.089	0.088	0.089	0.089	0.089	0.089	0.089	0.089	0.086	0.085	0.068	0.068	0.053	0.052
	BSS	0.011		0.000		0.000		0.000		0.012		0.000		0.019	
t = 72	BS	0.112	0.111	0.112	0.112	0.112	0.111	0.113	0.112	0.110	0.108	0.094	0.093	0.078	0.076
	BSS	0.009		0.000		0.009		0.009		0.018		0.011		0.026	
t = 96	BS	0.126	0.125	0.126	0.126	0.126	0.125	0.126	0.125	0.124	0.122	0.108	0.107	0.092	0.091
	BSS	0.008		0.000		0.008		0.008		0.016		0.009		0.011	
t = 120	BS	0.156	0.155	0.156	0.156	0.156	0.155	0.156	0.156	0.155	0.153	0.140	0.139	0.124	0.122
	BSS	0.006		0.000		0.006		0.000		0.013		0.007		0.016	

Abbreviations: BS: Brier score; BSS: Brier skill score.

Simulations in the population with XDR-TB infection

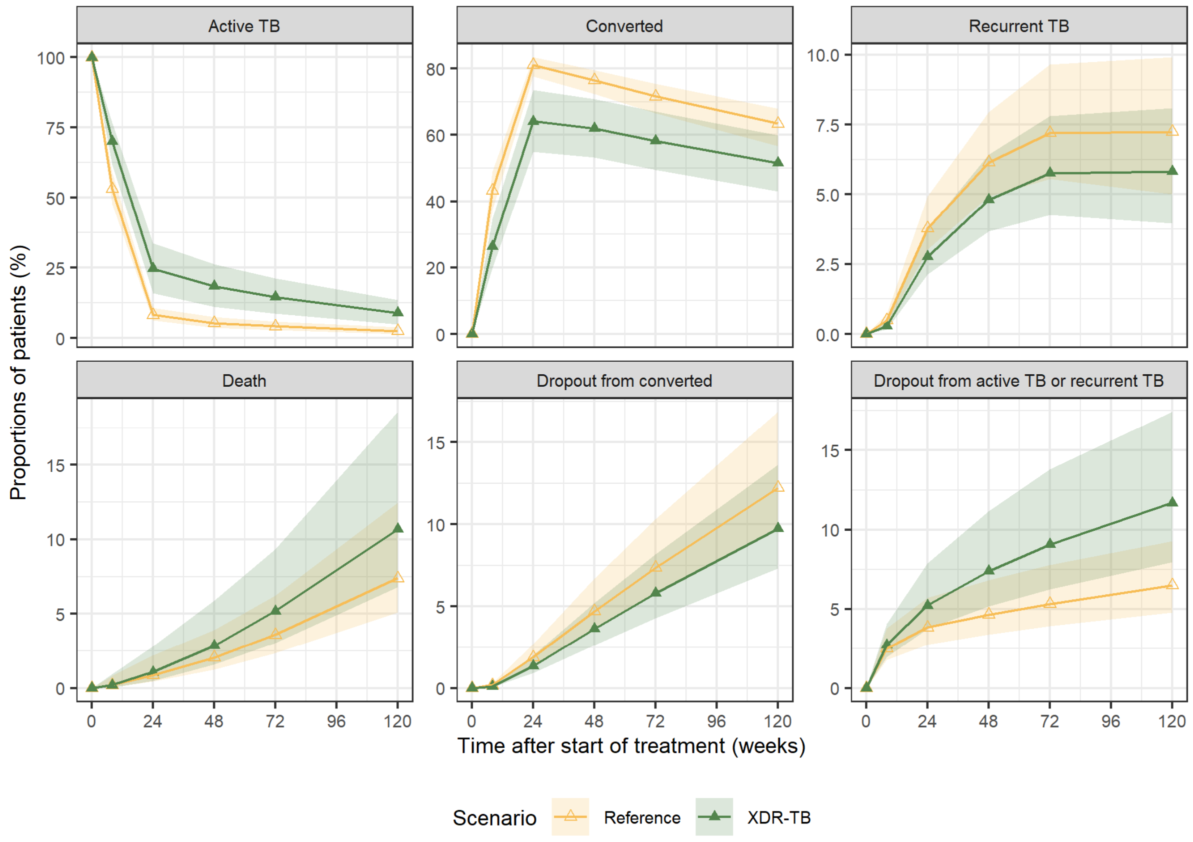

Figure S2. The proportions of patients with or without XDR-TB resistance having possible outcomes throughout the study. Lines with different symbols indicate the median of predicted proportions of patients in each state under reference or XDR-TB scenarios. Shaded areas indicate the 90% prediction intervals representing parameter estimate uncertainty. Patient population in the scenario XDR-TB was assumed to have XDR-TB resistance but typical values of other predictors as a reference individual.

NONMEM code

```
$PROBLEM  Multistate model for pulmonary tuberculosis
$INPUT    ID WEEK TIME CMT AMT EVID DV STATE C208 TYPE
          SEX AGE WT TBTYPE MTTP HL2 MMBLend
;Sim_start: add/remove for simulation
$DATA     data.csv IGNORE=@ IGNORE(TYPE.EQ.0)
;$DATA    data.csv IGNORE=@
;Sim_end
$SUBROUTINE ADVAN13 TOL=6
$MODEL   NCOMP=6 COMP=(S1) COMP=(S2) COMP=(S3) COMP=(S4) COMP=(S5)
COMP=(DUMMY,DEFOBS)
$PK
;-------------------
BSV     = ETA(1) ; place holder

IF(NEWIND.NE.2) XDV = 1        ; initial DV = 1
IF(NEWIND.NE.2) PREVDV = 1  ; previous DV

; Sim_start: add for simulation
IF(DV.GE.1) DV1 = DV
IF(DV.GE.1) XDV = DV
;Sim_end

;----- Covariates ------
;---median TTP at baseline
MTTP2 = MTTP
IF(MTTP2.EQ.-99) MTTP2 = 217.6667

;---MMBL at the end of treatment
FLAG_MMBL = 0 ; Flagging using MMBLend as a predictor after bedaquiline treatment
IF(WEEK.GT.26) FLAG_ MMBL = 1

MMBLend2 = LOG(MMBLend)

;---TB type
XDR = 0
IF(TBTYPE.EQ.4) XDR = 1

;----AGE
AGE2 = AGE
IF(AGE2.EQ.-99) AGE2 = 33

;----SEX
```

```
SEX2 = SEX
IF(SEX2.EQ.-99) SEX2 = 0

;----Baseline WT
basWT = WT
IF(basWT.EQ.-99) basWT = 55

;------------------- Mean transit times in weeks --------------
MTT23   = THETA(1)*24*7     ; From Converted to Recurrent TB
MTT32   = THETA(2)*24*7     ; From Recurrent TB to Converted
MTT14   = THETA(3)*24*7     ; From Active TB to Dropout
MTT24   = THETA(4)*24*7     ; From Converted to Dropout
MTT34   = MTT24             ; From Recurrent TB to Dropout
MTT15   = THETA(5)*24*7     ; From Active TB to Death
MTT25   = THETA(6)*24*7     ; From Converted to Death
MTT35   = MTT15             ; From Recurrent TB to Death

;------------------- Define surge function parameters --------------
SA = THETA(7)/10000*EXP(THETA(12)*(HL2-0.69443)+THETA(13)*XDR)            ;
Surge amplitude in hours
PT = THETA(8)*24*7*EXP(-THETA(11)*((MTTP2- 217.6667)/24/7)-THETA(12)*(HL2-
0.69443))            ; Peak time in weeks
SW = THETA(9)*24*7*EXP(THETA(11)*((MTTP2- 217.6667)/24/7))            ; Surge
width in weeks

;------------------- Define Weibull parameters --------------
;; Active infection to Death
HZL15 = 1/MTT15
HZA15 = THETA(10)

;--------- Initialize probabilities -----------
IF(NEWIND.NE.2) THEN
BIO1   = 1          ; at time 0, all individuals initialized with state 1
BIO2   = 0
BIO3   = 0
BIO4   = 0
BIO5   = 0
ENDIF
;-----------------------------------------------------
;--------- "Bioavailability" factors -------------------
 F1    = BIO1
 F2    = BIO2
 F3    = BIO3
 F4    = BIO4
```

```
 F5    = BIO5
;---------------------------------------------------------

$DES
HZ12= SA / (((T - PT) / SW)**2 + 1)

DEL = 1E-16
WB15 =  HZL15*HZA15*(HZL15*(T+DEL))**(HZA15-1)

;----------- Probability transfer constants -----------
K12 = HZ12
;--- include MMBLend only after the end of treatment
K23 = 1/MTT23*EXP(THETA(17)*SEX2+THETA(18)*FLAG_MMBL*(MMBLend2-
LOG(0.000055726)))
K32 = 1/MTT32
K14 = 1/MTT14*EXP(THETA(14)*C208+THETA(15)*(AGE2-33))
K24 = 1/MTT24*EXP(THETA(14)*C208+THETA(15)*(AGE2-33))
K34 = K24
K15 = WB15*EXP(THETA(16)*(basWT-55))
K25 = 1/MTT25*EXP(THETA(16)*(basWT-55))
K35 = K15

;--Differential equations for the probability of each state
DADT(1) = - A(1)*(K12 + K14 + K15)                              ; ACTIVE TB
DADT(2) =   A(1)*K12 +A(3)*K32 -A(2)*K23 -A(2)*K24 -A(2)*K25    ; CONVERTED
DADT(3) =   A(2)*K23 -A(3)*K32 -A(3)*K34 -A(3)*K35              ; RECURRENT TB
DADT(4) =   A(1)*K14 +A(2)*K24 +A(3)*K34                        ; DROPOUT
DADT(5) =   A(1)*K15 +A(2)*K25 +A(3)*K35                        ; DEATH
DADT(6) = 0                                          ; DUMMY PK Compartment

;-------------------------------------------------------
$ERROR

P_1 = A(1)  ; Probability of observing Active TB state
P_2 = A(2)  ; Probability of observing Converted state
P_3 = A(3)  ; Probability of observing Recurrent TB state
P_4 = A(4)  ; Probability of observing Dropout
P_5 = A(5)  ; Probability of observing Death

TOT = P_1 + P_2 + P_3 + P_4 + P_5  ; Check the sum of the probabilities is equal to 1
;-------------------------------------------------------
```

```
;---- Model prediction -----------------------------------
IF(DV.EQ.1.AND.EVID.EQ.0)  Y = P_1
IF(DV.EQ.2.AND.EVID.EQ.0)  Y = P_2
IF(DV.EQ.3.AND.EVID.EQ.0)  Y = P_3
IF(DV.EQ.4.AND.EVID.EQ.0)  Y = P_4
IF(DV.EQ.5.AND.EVID.EQ.0)  Y = P_1*K15+P_2*K25+P_3*K35 ; Observed death is an
actual date
;----------------------------------------------------------

; ---------Cumulative probabilities -----
CUP1 = A(1) ; 1
CUP2 = A(1) + A(2) ; 1 + 2
CUP3 = A(1) + A(2) + A(3) ; 1 + 2 + 3
CUP4 = A(1) + A(2) + A(3) + A(4) ; 1 + 2 + 3 + 4
CUP5 = A(1) + A(2) + A(3) + A(4) + A(5) ; 1 + 2 + 3 + 4 + 5 ; Check the sum of the
probabilities is equal to 1
;----------------------------------------------------------

; Sim_start: remove for simulation
;   IF(ICALL.EQ.4) THEN
;
;    IF (EVID.EQ.0) THEN
;        CALL RANDOM (2,R)
;        USUR=R
;
;       IF(USUR.LE.CUP1) XDV=1
;       IF(USUR.GT.CUP1.AND.USUR.LE.CUP2) XDV=2
;       IF(USUR.GT.CUP2.AND.USUR.LE.CUP3) XDV=3
;       IF(USUR.GT.CUP3.AND.USUR.LE.CUP4) XDV=4
;       IF(USUR.GT.CUP4) XDV=5
;
;    IF(PREVDV.EQ.4) XDV=4
;    PREVDV = XDV
;    ENDIF
;  ENDIF
;Sim_end

;--------- Update Bioavailability factors -------------------
BIO1=0
BIO2=0
BIO3=0
BIO4=0
BIO5=0
IF(XDV.EQ.1)  BIO1 = 1
```

```
IF(XDV.EQ.2)  BIO2 = 1
IF(XDV.EQ.3)  BIO3 = 1
IF(XDV.EQ.4)  BIO4 = 1
IF(XDV.EQ.5)  BIO5 = 1

$THETA
(0,295.375)        ; 1   MTT23
(0,61.8711)        ; 2   MTT32
(0,260.086)        ; 3   MTT14
(0,667.209)        ; 4   MTT24
(0,192.364)        ; 5   MTT15
(0,3803.44)        ; 6   MTT25
(0,11.2899)        ; 7   SA in hours-1
(0,11.3799)        ; 8   PT in week
(0,5.58883)        ; 9   SW in week
(0,1.96131)        ; 10  HZA15
0.442668           ; 11 Beta MTTP12
-0.686145          ; 12 Beta HL2_12
-0.622792          ; 13 Beta XDR12
0.909188           ; 14 Beta Study14_24
-0.0230092         ; 15 Beta Age14_24
-0.0838138         ; 16 Beta basWT15_25
-0.812999          ; 17 Beta SEX23
0.0371081          ; 18 Beta MMBLend_23
;---- multistate model parameters
$OMEGA  0  FIX
;Sim_start : add/remove for simulation
;$SIGMA  0  FIX
;$SIMULATION (1722475071) (1280753459 UNIFORM) (1972864618 UNIFORM)
ONLYSIM NSUBPROBLEMS=1000
$ESTIMATION METHOD=0 LIKE SLOW PRINT=1 SIGL=6 NSIG=2 MAXEVALS=9999
;Sim_end
$COVARIANCE
```

References

1. Diacon AH, Pym A, Grobusch MP *et al*. Multidrug-Resistant Tuberculosis and Culture Conversion with Bedaquiline. *N Engl J Med* 2014; **371**: 723–32.
2. Pym AS, Diacon AH, Tang SJ *et al*. Bedaquiline in the treatment of multidrug- and extensively drug-resistant tuberculosis. *Eur Respir J* 2016; **47**: 564–74.
3. Svensson EM, Svensson RJ, te Brake LHM *et al*. The Potential for Treatment Shortening With Higher Rifampicin Doses: Relating Drug Exposure to Treatment Response in Patients With Pulmonary Tuberculosis. *Clin Infect Dis* 2018; **67**: 34–41.
4. Chang V, Phillips PPJ, Imperial MZ, Nahid P, Savic RM. A comparison of clinical development pathways to advance tuberculosis regimen development. *BMC Infect Dis* 2022; **22**: 920.
5. Svensson EM, Dosne A, Karlsson MO. Population Pharmacokinetics of Bedaquiline and Metabolite M2 in Patients With Drug-Resistant Tuberculosis: The Effect of Time-Varying Weight and Albumin. *CPT Pharmacometrics Syst Pharmacol* 2016; **5**: 682–91.
6. Svensson EM, Karlsson MO. Modelling of mycobacterial load reveals bedaquiline's exposure–response relationship in patients with drug-resistant TB. *J Antimicrob Chemother* 2017; **72**: 3398–405.
7. Tanneau L, Karlsson MO, Svensson EM. Understanding the drug exposure–response relationship of bedaquiline to predict efficacy for novel dosing regimens in the treatment of multidrug-resistant tuberculosis. *Br J Clin Pharmacol* 2020; **86**: 913–22.
8. Beal S, Sheiner L, Boeckmann A *et al*. NONMEM 7.4 Users Guides (ICON plc, Gaithersburg, MD, 1989-2022) [Internet]. Available from: https://nonmem.iconplc.com/nonmem751. Accessed 8 September 2023.
9. Nordgren R, Freiberga S, Ueckert S *et al*. PsN: An open source toolkit for non-linear mixed effects modelling [Internet]. 2016. Available from: https://uupharmacometrics.github.io/PsN/. Accessed 8 September 2023.
10. R Core Team. R: A language and environment for statistical computing [Internet]. Vienna, Austria: R Foundation for Statistical Computing; 2022. Available from: https://www.r-project.org/. Accessed 8 September 2023.
11. Keizer R, Karlsson M, Hooker A. Modeling and Simulation Workbench for NONMEM: Tutorial on Pirana, PsN, and Xpose. *CPT Pharmacometrics Syst Pharmacol* 2013; **2**: 50.
12. Mouksassi S. Coveffectsplot: Produce forest plots to visualize covariate effects. R package version 1.0.2 [Internet]. 2022. Available from: https://CRAN.R-project.org/package=coveffectsplot. Accessed 8 September 2023.
13. Marier J, Teuscher N, Mouksassi M. Evaluation of covariate effects using forest plots and introduction to the *coveffectsplot* R package. *CPT Pharmacometrics Syst Pharmacol* **2022**; **11**: 1283–93.
14. Baron K. Mrgsolve: simulate from ODE-based models. R package version 1.0.6 [Internet]. 2022. Available from: https://CRAN.R-project.org/package=mrgsolve. Accessed 8 September 2023.
